# Relationships between Isometric Force-Time Characteristics and Dynamic Performance

**DOI:** 10.3390/sports5030068

**Published:** 2017-09-13

**Authors:** Thomas Dos’Santos, Christopher Thomas, Paul Comfort, John J. McMahon, Paul A. Jones

**Affiliations:** Directorate of Sport, Exercise & Physiotherapy, University of Salford, Salford, Greater Manchester M6 6PU, UK; c.thomas2@edu.salford.ac.uk (C.T.); p.comfort@salford.ac.uk (P.C.); j.j.mcmahon@salford.ac.uk (J.J.M.); P.A.Jones@salford.ac.uk (P.A.J.)

**Keywords:** peak force, time-specific force, isometric mid-thigh pull, countermovement jump, squat jump, reactive strength index modified, power clean

## Abstract

The purpose of this study was to explore the relationships between isometric mid-thigh pull (IMTP) force-time characteristics (peak force and time-specific force vales (100–250 ms)) and dynamic performance and compare dynamic performance between stronger and weaker athletes. Forty-three athletes from different sports (rowing, soccer, bicycle motocross, and hockey) performed three trials of the squat jump (SJ), countermovement jump (CMJ), and IMTP, and performed a one repetition maximum power clean (PC). Reactive strength index modified (RSImod) was also calculated from the CMJ. Statistically significant large correlations between IMTP force-time characteristics and PC (*ρ* = 0.569–0.674, *p* < 0.001), and moderate correlations between IMTP force-time characteristics (excluding force at 100 ms) and RSImod (*ρ* = 0.389–0.449, *p* = 0.013–0.050) were observed. Only force at 250 ms demonstrated a statistically significant moderate correlation with CMJ height (*ρ* = 0.346, *p* = 0.016) and no statistically significant associations were observed between IMTP force-time characteristics and SJ height. Stronger athletes (top 10) demonstrated statistically significantly greater CMJ heights, RSImods, and PCs (*p* ≤ 0.004, *g* = 1.32–1.89) compared to weaker (bottom 10) athletes, but no differences in SJ height were observed (*p* = 0.871, *g* = 0.06). These findings highlight that the ability to apply rapidly high levels of force in short time intervals is integral for PC, CMJ height, and reactive strength.

## 1. Introduction

Maximum strength is an integral quality unpinning athletic performance [1,2,3], thus, assessing and monitoring the maximum force production qualities of athletes is of great importance to sports scientists and strength and conditioning coaches. One repetition maximum testing (1RM) of exercises such as the back squat, deadlift, and power clean (PC) are commonly used by practitioners to assess the maximum strength capabilities in athletic populations [2,4,5] and are used to monitor the effectiveness of training and inform future training. Although 1RM testing has demonstrated high reliability [6,7,8] and the maximal loads lifted are often used as a reference to prescribe training loads for future training, several limitations exist including duration of testing, technical competency, fatigue, and risk of injury [9,10]. An assessment which reduces these limitations and provides greater insight into the maximal and rapid force production capabilities of athletes is the isometric mid-thigh pull (IMTP).

Valuable information can be attained from the force-time curves recorded from IMTPs, with peak force (PF) commonly evaluated [8,11,12,13]. Importantly, PF has been shown to be highly reliable within and between sessions [8,11,14,15,16] and may offer a surrogate to 1RM testing due to its strong associations with 1RM back squat [2,12,17,18,19], PC [17,18,20], deadlift [9], and weightlifting performance [13,21,22]. Furthermore, strong associations have been observed between absolute or relative PF with dynamic performance such as vertical jump (VJ) height [19,23,24,25,26,27,28], drop jump reactive strength index [29], sprint time [3,25,30], change of direction speed time [2,3,31,32], sprint cycling performance [23], and throwing distance [33,34]. Collectively, these findings rationalize the assessment of PF through IMTP testing and highlight the importance of PF to dynamic athletic performance.

While maximum strength is critical for athletics tasks, the ability to produce rapid and high levels of force in short time intervals is also integral for athletic performance [35,36,37]. IMTP testing permits the inspection of time-specific force values and rate of force development (RFD) during critical time intervals (50–300 ms) [11,13,26,38] similar to the contraction times and ground contact times of sprinting, jumping, changing direction, cycling, and striking [35,36,39]. Mixed findings have been reported between time-specific force values and dynamic performance. For example, greater IMTP time-specific force values have been associated with baseball batting performance [39], loaded VJ height [26], unloaded VJ height [25,26], and weightlifting performance [13]. West et al. [25] observed a strong and statistically significant inverse relationship between IMTP absolute and relative force at 100 ms and 10 m sprint time in professional rugby players, whereas, Northeast et al. [30] demonstrated no statistically significant relationship between IMTP force at 100 ms and sprint time in professional soccer players. Wang et al. [12] reported a strong positive relationship between IMTP time-specific force values (90–250 ms) and 1RM back squat in male rugby players, however, IMTP time-specific force values demonstrated no statistically significant relationships with 1RM deadlift in a recent study [9]. Moreover, Leary et al. [40] observed a moderate relationship between IMTP force at 150 ms and mean and maximum club head speed during a golf swing, although it is worth noting that these correlations approached statistical significance (*r* = 0.46–0.47, *p* = 0.06–0.07). A limited number of studies have examined the relationship between IMTP time-specific force values and VJ height [25,26], and no study to our knowledge has examined the relationship between IMTP time-specific force values and 1RM PC. As such, further research is required exploring the relationships between IMTP time-specific force values and dynamic tasks such as the PC and VJ.

Of interest to sports scientists and strength and conditioning coaches is the relationship between isometric force-time characteristics and countermovement jump (CMJ) height, CMJ peak power (PP), and CMJ peak RFD, in order to strengthen the rationale for strength diagnostic profiling of athletes through the IMTP. Numerous studies have shown a positive relationship between IMTP absolute PF and CMJ height [19,20,23,24,27], IMTP relative PF and CMJ height [25,26,28], IMTP absolute PF and CMJ PP [20,21,23,27], and IMTP absolute PF with CMJ peak RFD [27]. In addition, positive associations have been observed between IMTP time-specific force values and CMJ height [25,26] and CMJ PP [25]. Reactive strength index modified (RSImod), defined as jump height divided by time to take off (TTT) [41], is a metric easily obtainable from the CMJ when performed on a force platform and is suggested to provide an indication of an athlete’s slow stretch shortening cycle capabilities and ability to apply force quickly [42]. However, the relationship between IMTP force-time characteristics and RSImod from the CMJ has received very little attention and thus requires further research.

Reactive strength is the ability of the musculotendinous unit to produce a powerful concentric contraction following a rapid eccentric contraction (i.e., plyometric) [43] and is an essential quality for time contained plyometric athletic tasks such as sprinting, changing direction, and jumping [29,43,44,45]. RSImod is particularly important in jumping sports; for example, if two athletes with similar jump heights jump for a ball, the individual who can perform the movement quicker may have an advantage. To our knowledge only one study has examined the relationship between IMTP force-time characteristics and RSImod and reported moderate to large relationships between IMTP PF, force at-, and RFD and impulse over 200 ms with CMJ RSImod (*r* = 0.425–0.509, *p* < 0.05) [46]. However, it is worth noting that the IMTP force-time characteristics (excluding PF) investigated by Beckham et al. [46] were only investigated at and over 200 ms. Thus, further research is necessary investigating the relationship between IMTP force at different time epochs and RSImod to confirm if IMTP force-time characteristics provide an indication of an athlete’s reactive strength, given the importance of reactive strength for dynamic tasks such as sprinting, changing direction, and jumping [29,43,44,45].

The majority of IMTP correlation studies have possessed relatively low sample sizes (*n* = 5–19), making overall conclusions difficult [2,3,12,13,17,20,21,24,27,28,47]. Subsequently, there is a requirement, therefore, for further research to support the concept that IMTP force-time characteristics provide indications of dynamic performance (PC and VJ) and reactive strength. Thus, the aims of this study were to explore the relationships between IMTP force-time characteristics and dynamic performance (1RM PC, VJ height, and CMJ RSImod) in a large sample size and compare dynamic performance between stronger and weaker athletes. It was hypothesized that greater 1RM PCs, VJ heights, and RSImod would be associated with greater IMTP PFs and IMTP time-specific force values, and that stronger athletes (IMTP PF) would demonstrate superior dynamic performance compared to weaker athletes.

## 2. Materials and Methods

### 2.1. Subjects

Athletes (*n* = 43 (male *n* = 36, female *n* = 7), age: 19.7 ± 2.1 years, height: 1.72 ± 0.17 m, mass: 70.1 ± 12.4 kg) from rowing, soccer, bicycle motocross, and hockey participated in this study. The investigation was approved by the institutional ethics review board (HSCR16/36), and all subjects were informed of the benefits and risks of the investigation prior to signing an institutionally approved consent document to participate in the study. The study conformed to the principles of the World Medical Association’s Declaration of Helsinki. Subjects were familiar with all testing procedures and possessed 6–12 months resistance training experience of the PC and its derivatives. All testing was conducted by certified strength and conditioning specialists. At the time of testing subjects had just completed a 4-week maximum strength mesocycle and were in the middle of their competitive seasons, respectively.

### 2.2. Procedures

This study used a cross-sectional design where all subjects performed SJ, CMJ, IMTP, and 1RM PC over one testing session to examine the relationships between isometric force-time characteristics and dynamic performance. The procedures documented in this study were performed in accordance to previous investigations from our laboratory, which have demonstrated excellent reliability for IMTP force-time characteristics (Intraclass correlation coefficient (ICC) ≥ 0.86, coefficient of variation (CV) ≤ 7.9%) and VJ variables (ICC ≥ 0.89, CV ≤ 5.7%) [8,48,49]. On arrival, all participants had their height (Stadiometer; Seca, Birmingham, United Kingdom) and body mass assessed (Seca Digital Scales, Model 707), measured to the nearest 0.1 kg and 0.1 cm, respectively. All participants performed a standardized dynamic warm up consisting of 10 body weight squats, 10 lunges per leg, and three practice SJs and CMJs. Three trials were performed for the SJ, CMJ, and IMTP, with five minutes rest between tests, respectively.

### 2.3. Vertical Jump Testing

Both the SJ and CMJ trials were performed with the subjects standing on a force platform (type: 9286AA, dimensions 600 mm × 400 mm, Kistler Instruments Inc., Amherst, NY, USA) sampling at 1000 Hz, interfaced with a laptop computer running Bioware software (version 5.11, Kistler Instruments Inc., Amherst, NY, USA). Subjects were instructed to stand still for the initial one second of the data collection period (known as the silent period immediately prior to performing the jumps) [50] to allow for the subsequent determination of body weight. The raw, unfiltered, vertical force-time data for each jump trial were exported as text files and analyzed, in line with previous recommendations to minimize sources of error [51], using a customized Microsoft Excel spreadsheet (version 2016, Microsoft Corp., Redmond, WA, USA).

All jumps were performed whilst the subjects kept their hands on their hips, with any jumps that were inadvertently performed with the removal of their hands from their hips or the inclusion of arm swing omitted, and additional trials were performed after one minute of rest. For the SJ, subjects were instructed to squat down to a self-selected depth (approximately 90°), pause for a count of three, and then jump as “fast and as high as possible”, without performing any preparatory countermovement. Resultant force-time data were visually inspected to determine if any countermovement had been performed, and if it had, subjects repeated the trial after one minute of rest. Subsequent analysis of the SJ force-time data revealed that no trial exceeded the threshold used to determine a countermovement (five times the standard deviation of body weight, as derived during the silent period) [41,50], as described below. For the CMJ, subjects were instructed to jump as “fast and as high as possible”, performing a rapid dip, to a self-selected depth, which they believed would achieve their greatest jump height.

The start of the jumps were identified in line with current recommendations where the onset of movement for each jump trial was considered to have occurred 30 milliseconds prior to the instant when vertical force had reduced (CMJ) or increased (SJ) by five times the standard deviation of body weight, as derived during the silent period [41,50]. Instantaneous centre of mass (COM) velocity was calculated by dividing vertical force (excluding body weight) by body mass and then integrating the product using the trapezoid rule. The instant of take-off was defined as the instant in time when vertical force was less than five times the standard deviation of the first 300 milliseconds of the flight phase of the jump (the residual force when the subject is airborne, i.e., when the force platform was unloaded) following the onset of movement [41,52]. TTT was calculated as the duration between the onset of the movement and the instant of take-off [53]. Jump height was derived from vertical velocity at take-off [41]. RSImod was calculated as CMJ height divided by TTT [41]. The means of three trials were used for statistical analysis.

### 2.4. Isometric Mid-Thigh Pull

The IMTP testing was performed on a portable force plate sampling at 1000 Hz (type: 9286AA, dimensions 600 mm × 400 mm, Kistler Instruments Inc., Amherst, NY, USA) using a portable IMTP rack (Fitness Technology, Adelaide, Australia). Sampling at 1000 Hz has been shown to produce high reliability for isometric force-time variables [11]. A cold rolled steel bar was positioned to correspond to the athlete’s second-pull power clean position just below the crease of the hip [13]. The bar height could be adjusted (3 cm increments) at various heights above the force plate to accommodate different sized athletes. Athletes were strapped to the bar in accordance to previous research [21] and positioned in their self-selected mid-thigh clean position [15] (knee angle of 135–145° and a hip angle of 140–150°) established in the familiarization trials whereby feet were shoulder width apart, knees were flexed over the toes, shoulders were just behind the bar, and torso was upright [11,54]. All subjects received standardized instructions to pull as “fast and as hard as possible and push their feet directly into the force plate” until being told to stop, as these instructions have been shown to produce optimal results [55,56]. Once the body was stabilized (verified by watching the subject and force trace) the IMTP was initiated with the countdown “3, 2, 1, pull,” with subjects ensuring that maximal effort was applied for five seconds. Ground reaction force data were collected for a duration of eight seconds from the portable force platform which was interfaced with a laptop and recorded using Bioware software (Version 5.11; Kistler Instrument Corporation, Winterthur, Switzerland). Minimal pre-tension was allowed to ensure there was no slack in the body prior to initiation of pull and subjects were instructed to be as still as possible during the weighing period, without initiating a pull on the bar, until given the instructions to ‘pull’. Strong verbal encouragement was given for all trials and subjects. Trials without a stable baseline force trace during the weighing period (change in force > 50 N) were rejected along with trials with a countermovement (decrease in body weight > 50 N); in the case of a rejected trial, another trial was subsequently performed [57,58]. Two-minute rest periods were provided between trials.

All force-time data recorded during the IMTP were inspected using a using a customized Microsoft Excel spreadsheet (version 2016, Microsoft Corp., Redmond, WA, USA) to determine specific force-time characteristics. The maximum force generated during the five second maximum effort IMTP was reported as the absolute PF [14]. Additionally, time-specific force values at 100 ms (Force_100_), 150 ms (Force_150_), 200 ms (Force_200_), and 250 ms (Force_250_) were calculated [14]. The onset of the pull was determined when vertical ground-reaction force deviated >5% from the average body weight during the weighing period [58]. The combined residual force and body weight were calculated as the average force over a 1 second stationary weighing period (in mid-thigh pull position posture) prior to the initiation of the IMTP [58]. The force plate was zeroed between each trial when participants stood off the force plate, thus all force-time variables included body weight. The means of three trials were used for statistical analysis.

### 2.5. 1RM Power Clean

1RM PCs were performed after the IMTPs. Participants warmed up by performing the exercises with submaximal loads that they were accustomed to from their normal training, in accordance to National Strength and Conditioning Association (NSCA) 1RM protocol [59]. Participants were permitted a maximum of six progressively increasing loads, although all participants achieved their 1RM within four to five attempts. If athletes caught the bar below a 90° knee angle, this was classed as a no lift and the lift was performed again after a 3-minute rest period. Verbal encouragement was provided throughout maximal testing, with the technical feedback regarding the depth of the catch for the power clean. All testing was performed using standardized barbells, weights (Werksan weights and Olympic bar; Werksan, Moorestown, NJ, USA), and powerlift lifting platforms (Jefferson, IA, USA).

### 2.6. Statistical Analysis

Mean ± standard deviation (SD) were calculated for all variables. Within-session reliability was assessed via ICCs and CV was calculated as SD/mean × 100 between repeated trials on a per-person basis and the average for the sample was reported [60]. Minimum acceptable reliability was determined with an ICC > 0.7 and CV < 10% [60]. Normality was inspected for all variables using a Shapiro Wilks-test. CMJ variables, SJ height, and PC performance were normally distributed, however, all IMTP force-time characteristics were not normally distributed. As the aim was to examine the relationship between IMTP force-time characteristics and dynamic performance, Spearman’s rank correlations were performed. Correlations were evaluated using Hopkins’ scale [61]. In addition, subjects were divided into stronger and weaker groups based on absolute PF. Subjects above the upper 25th percentile were assigned to the stronger group and those below the lower 25th percentile were assigned to the weaker group in accordance to previous research [29]. Independent sample t-tests and Hedges’ *g* effect sizes [62] were implemented due to the small sizes of each group to assess the magnitude of differences in dynamic performance and IMTP force-time characteristics between stronger and weaker groups; effect sizes were interpreted using Hopkins’ scale [63]. The criterion for significance was set at *p* ≤ 0.05. To control for type 1 error for multiple correlations and familywise error for multiple t-tests, *p* values were Bonferroni corrected. All statistical analyses were performed using SPSS (version 23, IBM, New York, NY, USA).

## 3. Results

High ICCs (ICC = 0.921–0.992) and low levels of variance (CV = 3.7–8.0%) were observed for IMTP force-time characteristics, CMJ variables, and SJ height, with all variables meeting minimum acceptable reliability criteria (Table 1). Scatter plots for statistically significant relationships are presented in Figures S1–S3.

### 3.1. Relationships between IMTP Force-Time Characteristics and Dynamic Performance

- Statistically significant and large correlations were observed between all IMTP force-time characteristics and absolute PC (*ρ* = 0.569–0.674, *p* < 0.001) (Table 2).
- Statistically significant and moderate correlations were revealed between IMTP PF, Force_150_, Force_200_, and Force_250_ and RSImod (*ρ* = 0.389–0.449, *p* = 0.013–0.050) (Table 2).
- Only Force_250_ demonstrated a statistically significant and moderate correlation with CMJ height (*ρ* = 0.346, *p* = 0.016). No statistically significant associations were observed between IMTP force-time characteristics and SJ height (Table 2).

### 3.2. Dynamic Performance and IMTP Force-Time Characteristics Comparisons between Stronger and Weaker Athletes

- Stronger athletes demonstrated statistically significantly greater CMJ heights, RSImods, absolute PCs, and relative PCs compared to weaker athletes (*p* ≤ 0.004), with large effect sizes (*g* = 1.32–1.89) (Table 3).
- Very large and statistically significant differences were observed in IMTP PF and time-specific force values between stronger and weaker athletes (*p* < 0.0001, *g* = 2.33–3.89) (Table 3).
- Trivial and no statistically significant differences were found in SJ height and CMJ TTT between stronger and weaker athletes (*p* ≥ 0.742, *g* ≤ −0.14) (Table 3).

## 4. Discussion

The aims of the present study were to explore the relationships between IMTP force-time characteristics and dynamic performance (1RM PC, VJ height, and RSImod) in a large sample size, and to compare dynamic performance between stronger and weaker athletes. The results from this study revealed IMTP force-time characteristics were statistically significantly correlated to 1RM PC and RSImod, whereas Force_250_ was the only variable to statistically significantly correlate to CMJ height. Conversely, no statistically significant relationships were observed between IMTP force-time characteristics and SJ height (Table 2). Furthermore, statistically significantly greater 1RM PCs, CMJ heights, and RSImods were demonstrated by stronger athletes in contrast to weaker athletes, with large effect sizes observed (Table 3). In addition, very large differences in IMTP force-time characteristics were also found (Table 3), in agreement with the hypotheses. Collectively, these findings indicate the importance of maximum force and rapid force production over short time intervals for dynamic tasks such as the PC, CMJ, and reactive strength.

The present study observed a large and statistically significant correlation between IMTP PF and 1RM PC (*ρ* = 0.674, *p* < 0.001) which substantiates the results of previous research [17,18,20]. This finding is unsurprising due to the specificity of the IMTP modelled on the second pull position of the clean whereby the largest forces are generated [47,64]. A unique observation from this study was that statistically significant and large correlations were demonstrated between time-specific force values (100–250 ms) and 1RM PC (*ρ* = 0.569–0.659, *p* < 0.001). These results are similar to those by Beckham et al. [13], who observed strong correlations between time-specific force values (100–250 ms) and 1RM snatch, and the clean and jerk (*r* = 0.64–0.80, *p* < 0.05). High levels of force, power, and velocity in durations of 0.10–0.26 seconds have been reported during the second pull of the snatch and the clean [64,65], which likely explains the strong relationships observed in the present study between IMTP time-specific force values and 1RM PC performance (Table 2). Consequently, the results of this study establish the importance of PF and rapid force production in short time intervals for 1RM PC performance and suggest that IMTP force-time characteristics provide an indication of 1RM PC.

Contrary to our hypothesis, Force_250_ was the only IMTP variable to demonstrate a statistically significant relationship with CMJ height (*ρ* = 0.346, *p* = 0.016) (Table 2). This result contrasts with studies that have observed strong relationships between PF and CMJ height [19,23,24,27], however, it supports the findings of Kraska et al. [26] who observed positive relationships between time-specific force values and loaded and unloaded CMJ height (*r* = 0.34–0.54, *p* < 0.05). West et al. [25] only examined IMTP force at 100 ms but demonstrated a positive relationship between relative Force_100_ and CMJ height (*r* = 0.43, *p* < 0.01). A possible explanation for the positive relationship observed between Force_250_ and CMJ height in the present study could be due to the similarities in concentric phase durations which have been reported during CMJs (0.25–0.28 seconds) [52] and since concentric impulse has been identified as the key determinant of CMJ height [41,52]. Therefore, the ability to apply high levels of force in time intervals similar to the concentric phase durations of CMJs should be a distinguishing factor leading to superior CMJ height.

Substantiating the findings of Beckham et al. [46], moderate and statistically significant relationships between IMTP PF, Force_150_, Force_200_, and Force_250_ with CMJ RSImod (*ρ* = 0.389–0.449, *p* = 0.013–0.050) were observed. In addition, stronger athletes compared to weaker athletes demonstrated greater CMJ heights in similar TTTs, thus leading to greater RSImods (Table 3). Concentric forces and impulses have been reported to correlate with RSImod from CMJs [49], while moderate to very large relationships have also been demonstrated between CMJ RFD, CMJ PP, and CMJ PF with CMJ RSImod in male and female collegiate athletes [53]. Therefore, although beyond the scope of the present study, stronger athletes must have had a greater ability to generate concentric forces and impulses leading to greater CMJ heights. However, further research is required to explore the influence of strength on the kinetic and kinematic mechanisms underpinning CMJ and RSImod performance.

Contrary to expectations, no statistically significant relationships were observed between IMTP force-time characteristics and SJ height and no statistically significant differences in SJ heights were demonstrated between stronger and weaker athletes (Table 2 and Table 3). This finding corroborates Thomas et al. [66] who also demonstrated no statically significant relationships between IMTP PF and SJ height in a mixed sporting sample. Conversely, the findings of the present study contrast with previous studies that observed positive relationships between IMTP PF and SJ height [23,24,27], however, this discrepancy could be attributed to the present study investigating a heterogonous cohort of athletes (rowing, soccer, bicycle motocross, hockey) compared to the homogenous samples of cyclists [23], rugby players [5], mixed martial arts competitors [67], weightlifters [27], and surfers [24], respectively. It is also worth noting that the instructions for the vertical jumps may not have been optimal for maximizing jump height [68]. For example, Talpey et al. [68] recently found that the instruction to “jump as high as possible” produced greater jump squat heights (~4%) compared to the instructions “fast leg extension” (*p* < 0.05). However, a shortcoming of the abovementioned study was that TTT was not examined, thus, the subsequent impact on RSImod could not be determined. Talpey et al. [68] observed a greater countermovement depth with the “jump as high as possible” instruction which would likely indicate a longer TTT in comparison to “fast leg extension”. As the present study aimed to examine both CMJ height and RSImod, and the fact that RSImod is both influenced by TTT and jump height, it could be argued that the instructions to “jump as fast and as high possible” in accordance to previous research [41,48,49,52] were essential to achieve better RSImods. However, further research is necessary to explore the impact of different verbal instructions on RSImod from the CMJ.

The present study compared dynamic performance between stronger and weaker athletes based on absolute PF (Table 3), similar to previous studies in sprint cyclists [23], netballers [69], and collegiate athletes from multiple sports [26,29]. Stronger athletes in the present study demonstrated statistically significantly greater CMJ heights compared to weaker athletes (*p* = 0.007, *g* = 1.32), which was comparable to the findings of previous studies that observed greater CMJ heights in stronger netballers [69] and collegiate athletes [26] compared to their weaker counterparts, with both studies reporting a moderate effect size (*d* = 0.60–1.00). Additionally, corroborating the observations of Beattie et al. [29], stronger collegiate athletes displayed statistically significantly (*p* = 0.005, *g* = 1.36) greater reactive strength (RSImod) compared to weaker athletes. Furthermore, the results of the current study revealed stronger athletes displayed both superior PC performance (*p* ≤ 0.004, *g* = 1.43–1.89) and absolute PF and time-specific force values (*p* < 0.001, *g* = 2.33–3.89), thus substantiating the findings by Kraska et al. [26] who also documented greater time-specific force values (90–250 ms) (*p* ≤ 0.003, *d* = 2.68–3.54) in stronger collegiate athletes. Taken together, strength and conditioning coaches should consider developing their athletes’ expression of force through strength and power training, in light of the findings from the present study and previous research reporting stronger athletes demonstrate superior dynamic performance.

A limitation of the present study was that vertical sagittal plane movements such as the PC and VJs were only examined, however, further research is required to explore the relationships between isometric force-time characteristics with horizontal linear speed and multiplanar movements such as change of direction speed tasks. Moreover, the present study only examined the relationship between IMTP force-time characteristics and VJ height, and RSImod, with no inspection of kinetic and kinematic variables such as power, impulse, force, and velocity during the VJs. Future research should compare VJ kinetic and kinematics between stronger and weaker athletes to attain a greater understanding of the mechanisms underpinning superior VJ performance. Nonetheless, within the context of these limitations, this study found stronger athletes demonstrated greater 1RM PCs, CMJ heights, and RSImods compared to weaker athletes (Table 3). Furthermore, statistically significant and positive associations were observed between IMTP force-time characteristics and 1RM PC, RSImod, while Force_250_ correlated to CMJ height (Table 2).

## 5. Conclusions

The results from this study revealed large and statistically significant relationships between IMTP force-time characteristics and 1RM PC, and moderate relationships with CMJ RSImod, whereas Force_250_ was the only variable to demonstrate a statistically significant correlation with CMJ height. Additionally, stronger athletes displayed superior 1RM PCs, CMJ heights, and RSImods in comparisons to weaker athletes, potentially highlighting that the ability to apply high and rapid levels of force in short time intervals is integral for dynamic performance during the PC and CMJ, and that it is essential for reactive strength. Therefore, sports scientists and strength and conditioning coaches should consider monitoring their athletes’ strength characteristics with the IMTP given the relationships with 1RM PC, RSImod, and CMJ height. Strength and conditioning coaches should consider developing their athletes’ expression of force through strength and power training due to the positive associations with dynamic performance.

## Figures and Tables

**Table 1:** Descriptive statistics and reliability measures for IMTP, CMJ, SJ and PC variables.

Assessment	Variable	Mean	SD	ICC	CV (%)
**IMTP**	PF (N)	2441	647	0.992	3.7
	Force_100_ (N)	1308	344	0.961	7.3
	Force_150_ (N)	1593	464	0.965	8.0
	Force_200_ (N)	1820	489	0.965	6.8
	Force_250_ (N)	1917	488	0.968	6.2
**CMJ**	Height (m)	0.32	0.05	0.979	3.7
	RSImod (ratio)	0.45	0.09	0.943	7.2
	TTT (s)	0.74	0.11	0.921	4.0
**SJ**	Height (m)	0.29	0.06	0.971	4.9
**PC**	Absolute (kg)	64.6	16.7		
	Relative (N∙kg^−1^)	0.92	0.15		

Key: IMTP: Isometric mid-thigh pull; CMJ: Countermovement jump; SJ: Squat jump; PC: Power clean; Force100: Force at 100 ms; Force150: Force at 150 ms; Force200: Force at 200 ms; Force250: Force at 250 ms; RSImod: Reactive strength index modified; TTT: Time to take off; BM: Body mass; ICC: Intraclass correlation coefficient; CV: Coefficient of variation.

**Table 2:** Spearman’s correlations between IMTP force-time characteristics and dynamic performance.

Assessment	Correlation and *p* Value	SJ Height	CMJ Height	RSImod	PC
PF	*ρ*	0.216	0.441	0.389 *	0.674 **
	*p* value	1.000	0.146	0.050	<0.001
Force_100_	*ρ*	0.085	0.333	0.362	0.633 **
	*p* value	1.000	0.269	0.086	<0.001
Force_150_	*ρ*	0.034	0.296	0.426 *	0.569 **
	*p* value	1.000	0.130	0.022	<0.001
Force_200_	*ρ*	0.078	0.339	0.449 *	0.629 **
	*p* value	1.000	0.114	0.013	<0.001
Force_250_	*ρ*	0.082	0.346 *	0.416 *	0.659 **
	*p* value	0.818	0.016	0.028	<0.001

Key: CMJ: Countermovement jump; SJ: Squat jump; RSImod: Reactive strength index modified; PC: Power clean; Force_100_: Force at 100 ms; Force_150_: Force at 150 ms; Force_200_: Force at 200 ms; Force_250_: Force at 250 ms; * *p* ≤ 0.05; ** *p* ≤ 0.01.

**Table 3:** Strong vs. weak comparisons for isometric force-time characteristics and dynamic performance.

Assessment and Variable		Strong (*N* = 10)		Weak (*N* = 10)		*p*	*g*	Descriptor
		Mean	SD	Mean	SD			
IMTP	PF (N)	3370	547	1758	128	<0.0001	3.89	Very Large
	Relative PF (N∙kg^−1^)	44.0	4.9	30.4	3.3	<0.0001	3.11	Very Large
	Force_100_ (N)	1685	309	1058	194	<0.0001	2.33	Very Large
	Force_150_ (N)	2181	406	1269	200	<0.0001	2.73	Very Large
	Force_200_ (N)	2474	443	1427	212	<0.0001	2.89	Very Large
	Force_250_ (N)	2573	439	1460	164	<0.0001	3.22	Very Large
CMJ	Height (m)	0.37	0.06	0.29	0.05	0.007	1.32	Large
	RSImod (ratio)	0.53	0.09	0.41	0.07	0.005	1.36	Large
	TTT (s)	0.71	0.10	0.72	0.10	0.742	−0.14	Trivial
SJ	Height (m)	0.29	0.08	0.30	0.06	0.871	−0.06	Trivial
PC	Absolute (kg)	81.2	17.0	49.5	15.0	<0.0001	1.89	Large
	Relative (N∙kg^−1^)	1.05	0.16	0.83	0.14	0.004	1.43	Large

Key: IMTP: Isometric mid-thigh pull; CMJ: Countermovement jump; SJ: Squat jump; PC: Power clean; Force_100_: Force at 100 ms; Force_150_: Force at 150 ms; Force_200_: Force at 200 ms; Force_250_: Force at 250 ms; RSImod: Reactive strength index modified; TTT: Time to take off; BM: Body mass.

## Supplementary Materials

The following are available online at www.mdpi.com/2075-4663/5/3/68/s1, Figures S1–S3: Spearman’s correlations between IMTP force-time characteristics and dynamic performance.

## References

[1] Suchomel T.J., Nimphius S., Stone M.H. (2016). The importance of muscular strength in athletic performance. Sports Med..

[2] Spiteri T., Nimphius S., Hart N.H., Specos C., Sheppard J.M., Newton R.U. (2014). The contribution of strength characteristics to change of direction and agility performance in female basketball athletes. J. Strength Cond. Res..

[3] Thomas C., Comfort P., Chiang C.-Y., Jones P. (2015). Relationship between isometric mid-thigh pull variables and sprint and change of direction performance in collegiate athletes. J. Trainol..

[4] Comfort P., Haigh A., Matthews M.J. (2012). Are changes in maximal squat strength during preseason training reflected in changes in sprint performance in rugby league players?. J. Strength Cond. Res..

[5] Baker D. (2001). The effects of an in-season of concurrent training on the maintenance of maximal strength and power in professional and college-aged rugby league football players. J. Strength Cond. Res..

[6] Comfort P., McMahon J.J. (2015). Reliability of maximal back squat and power clean performances in inexperienced athletes. J. Strength Cond. Res..

[7] Faigenbaum A.D., McFarland J.E., Herman R., Naclerio F., Ratamess N.A., Kang J., Myer G.D. (2012). Reliability of the one repetition-maximum power clean test in adolescent athletes. J. Strength Cond. Res..

[8] Dos’Santos T., Thomas C., Comfort P., McMahon J.J., Jones P.A., Oakley N.P., Young A.L. (2017). Between-session reliability of isometric mid-thigh pull kinetics and maximal power clean performance in male youth soccer players. J. Strength Cond. Res..

[9] De Witt J.K., English K.L., Crowell J.B., Kalogera K.L., Guilliams M.E., Nieschwitz B.E., Hanson A.M., Ploutz-Snyder L.L. (2016). Isometric mid-thigh pull reliability and relationship to deadlift 1rm. J. Strength Cond. Res..

[10] Drake D., Kennedy R., Wallace E. (2017). The validity and responsiveness of isometric lower body multi-joint tests of muscular strength: A systematic review. Sports Med. Open.

[11] Dos’Santos T., Jones P.A., Kelly J., McMahon J.J., Comfort P., Thomas C. (2016). Effect of sampling frequency on isometric mid-thigh pull kinetics. Int. J. Sports Physiol. Perform..

[12] Wang R., Hoffman J.R., Tanigawa S., Miramonti A.A., La Monica M.B., Beyer K.S., Church D.D., Fukuda D.H., Stout J.R. (2016). Isometric mid-thigh pull correlates with strength, sprint, and agility performance in collegiate rugby union players. J. Strength Cond. Res..

[13] Beckham G., Mizuguchi S., Carter C., Sato K., Ramsey M., Lamont H., Hornsby G., Haff G., Stone M. (2013). Relationships of isometric mid-thigh pull variables to weightlifting performance. J. Sports Med. Phys. Fitness.

[14] Haff G.G., Ruben R.P., Lider J., Twine C., Cormie P. (2015). A comparison of methods for determining the rate of force development during isometric midthigh clean pulls. J. Strength Cond. Res..

[15] Comfort P., Jones P.A., McMahon J.J., Newton R. (2015). Effect of knee and trunk angle on kinetic variables during the isometric mid-thigh pull: Test-retest reliability. Int. J. Sports Physiol. Perform..

[16] Dos’Santos T., Thomas C., Jones P.A., McMahon J.J., Comfort P. (2017). The effect of hip joint angle on isometric mid-thigh pull kinetics. J. Strength Cond. Res..

[17] McGuigan M.R., Winchester J.B., Erickson T. (2006). The importance of isometric maximum strength in college wrestlers. J Sports Sci. Med..

[18] McGuigan M.R., Winchester J.B. (2008). The relationship between isometric and dynamic strength in college football players. J Sports Sci. Med..

[19] McGuigan M.R., Newton M.J., Winchester J.B., Nelson A.G. (2010). Relationship between isometric and dynamic strength in recreationally trained men. J. Strength Cond. Res..

[20] Nuzzo J.L., McBride J.M., Cormie P., McCaulley G.O. (2008). Relationship between countermovement jump performance and multijoint isometric and dynamic tests of strength. J. Strength Cond. Res..

[21] Haff G.G., Carlock J.M., Hartman M.J., Kilgore J.L., Kawamori N., Jackson J.R., Morris R.T., Sands W.A., Stone M.H. (2005). Force--time curve characteristics of dynamic and isometric muscle actions of elite women olympic weightlifters. J. Strength Cond. Res..

[22] Stone M.H., Sands W.A., Pierce K.C., Carlock J.O.N., Cardinale M., Newton R.U. (2005). Relationship of maximum strength to weightlifting performance. Med. Sci. Sports Exerc..

[23] Stone M.H., Sands W.A., Carlock J.O.N., Callan S.A.M., Dickie D.E.S., Daigle K., Cotton J., Smith S.L., Hartman M. (2004). The importance of isometric maximum strength and peak rate-of-force development in sprint cycling. J. Strength Cond. Res..

[24] Secomb J.L., Farley O.R.L., Lundgren L., Tran T.T., King A., Nimphius S., Sheppard J.M. (2015). Associations between the performance of scoring manoeuvres and lower-body strength and power in elite surfers. Int. J. Sports Sci. Coach..

[25] West D.J., Owen N.J., Jones M.R., Bracken R.M., Cook C.J., Cunningham D.J., Shearer D.A., Finn C.V., Newton R.U., Crewther B.T. (2011). Relationships between force time characteristics of the isometric midthigh pull and dynamic performance in professional rugby league players. J. Strength Cond. Res..

[26] Kraska J.M., Ramsey M.W., Haff G.G., Fethke N., Sands W.A., Stone M.E., Stone M.H. (2009). Relationship between strength characteristics and unweighted and weighted vertical jump height. Int. J. Sports Physiol. Perform..

[27] Kawamori N., Rossi S.J., Justice B.D., Haff E.E., Pistilli E.E., O’Bryant H.S., Stone M.H., Haff G.G. (2006). Peak force and rate of force development during isometric and dynamic mid-thigh clean pulls performed at various intensities. J. Strength Cond. Res..

[28] Khamoui A.V., Brown L.E., Nguyen D., Uribe B.P., Coburn J.W., Noffal G.J., Tran T. (2011). Relationship between force-time and velocity-time characteristics of dynamic and isometric muscle actions. J. Strength Cond. Res..

[29] Beattie K., Carson B.P., Lyons M., Kenny I.C. (2016). The relationship between maximal-strength and reactive-strength. Int. J.Sports Physiol. Perform..

[30] Northeast J., Russell M., Shearer D., Cook C., Kilduff L. (2017). Predictors of linear and multidirectional acceleration in elite soccer players. J. Strength Cond. Res..

[31] Conlon J., Haff G.G., Nimphius S., Tran T., Newton R.U. (2013). Vertical jump velocity as a determinant of speed and agility performance. J. Aust. Strength Cond..

[32] Thomas C., Dos’Santos T., Comfort P., Jones P.A. (2016). Relationship between isometric strength, sprint, and change of direction speed in male academy cricketers. J. Trainol..

[33] Stone M.H., Sanborn K., O’Bryant H.S., Hartman M., Stone M.E., Proulx C., Ward B., Hruby J. (2003). Maximum strength-power-performance relationships in collegiate throwers. J. Strength Cond. Res..

[34] Whittington J., Schoen E., Labounty L., Hamdy R., Ramsey M., Stone M., Sands W., Haff G., Stone M. (2009). Bone mineral density and content of collegiate throwers: Influence of maximum strength. J. Sports Med. Phys. Fitness.

[35] Weyand P.G., Sternlight D.B., Bellizzi M.J., Wright S. (2000). Faster top running speeds are achieved with greater ground forces not more rapid leg movements. J. Appl. Physiol..

[36] Dos’Santos T., Thomas C., Jones A.P., Comfort P. (2017). Mechanical determinants of faster change of direction speed performance in male athletes. J. Strength Cond. Res..

[37] Newton R.U., Kraemer W.J. (1994). Developing explosive muscular power: Implications for a mixed methods training strategy. Strength Cond. J..

[38] Haff G.G., Stone M.H. (2015). Methods of developing power with special reference to football players. Strength Cond. J..

[39] Bailey C.A., Sato K., Hornsby W.G. (2013). Predicting offensive performance in collegiate baseball players using isometric force production characteristics. Chin.J. Sports Biomech..

[40] Leary B.K., Statler J., Hopkins B., Fitzwater R., Kesling T., Lyon J., Phillips B., Bryner R.W., Cormie P., Haff G.G. (2012). The relationship between isometric force-time curve characteristics and club head speed in recreational golfers. J. Strength Cond. Res..

[41] McMahon J.J., Rej S.J.E., Comfort P. (2017). Sex differences in countermovement jump phase characteristics. Sports.

[42] Suchomel T.J., Sole C.J., Bailey C.A., Grazer J.L., Beckham G.K. (2015). A comparison of reactive strength index-modified between six us collegiate athletic teams. J. Strength Cond. Res..

[43] Newton R.U., Laursen P., Young W. (2009). Clinical exercise testing and assessment of athletes. Encycl. Sports Med..

[44] Green B.S., Blake C., Caulfield B.M. (2011). A comparison of cutting technique performance in rugby union players. J. Strength Cond. Res..

[45] Schmidtbleicher D. (1992). Training for power events. Strength Power Sport.

[46] Beckham G.K., Suchomel T.J., Bailey C.A., Sole C.J., Grazer J.L., Sato K., Sands W.A., Mizuguchi S. (2014). The relationship of the reactive strength index-modified and measures of force development in the isometric mid-thigh pull. Proceedings of the 32th International Conference of Biomechanics in Sports.

[47] Haff G.G., Stone M., O’Bryant H.S., Harman E., Dinan C., Johnson R., Han K.-H. (1997). Force-time dependent characteristics of dynamic and isometric muscle actions. J. Strength Cond. Res..

[48] Comfort P., Thomas C., Dos’Santos T., Jones P.A., Suchomel T.J., McMahon J.J. (2017). Comparison of methods of calculating dynamic strength index. Int. J. Sports Physiol. Perform..

[49] McMahon J.J., Jones P.A., Suchomel T.J., Lake J., Comfort P. (2017). Influence of reactive strength index modified on force-and power-time curves. Int. J. Sports Physiol. Perform..

[50] Owen N.J., Watkins J., Kilduff L.P., Bevan H.R., Bennett M.A. (2014). Development of a criterion method to determine peak mechanical power output in a countermovement jump. J. Strength Cond. Res..

[51] Street G., McMillan S., Board W., Rasmussen M., Heneghan J.M. (2001). Sources of error in determining countermovement jump height with the impulse method. J. Appl. Biomech..

[52] McMahon J.J., Murphy S., Rej S.J.E., Comfort P. (2017). Countermovement jump phase characteristics of senior and academy rugby league players. Int. J. Sports Physiol. Perform..

[53] Suchomel T.J., Bailey C.A., Sole C.J., Grazer J.L., Beckham G.K. (2015). Using reactive strength index-modified as an explosive performance measurement tool in division i athletes. J. Strength Cond. Res..

[54] Dos’Santos T., Thomas C., Jones P.A., Comfort P. (2017). Assessing muscle strength asymmetry via a unilateral stance isometric mid-thigh pull. Int. J. Sports Physiol. Perform..

[55] Bemben M.G., Clasey J.L., Massey B.H. (1990). The effect of the rate of muscle contraction on the force-time curve parameters of male and female subjects. Res. Q. Exerc. Sport.

[56] Halperin I., Williams K.J., Martin D.T., Chapman D.W. (2016). The effects of attentional focusing instructions on force production during the isometric midthigh pull. J. Strength Cond. Res..

[57] Maffiuletti N.A., Aagaard P., Blazevich A.J., Folland J., Tillin N., Duchateau J. (2016). Rate of force development: Physiological and methodological considerations. Eur. J. Appl. Physiol..

[58] Dos’Santos T., Jones P.A., Comfort P., Thomas C. (2016). Effect of different onset thresholds on isometric mid-thigh pull force-time variables. J. Strength Cond. Res..

[59] Haff G.G., Triplett N.T. (2015). Essentials of Strength Training and Conditioning.

[60] Atkinson G., Nevill A.M. (1998). Statistical methods for assessing measurement error (reliability) in variables relevant to sports medicine. Sports Med..

[61] Hopkins W.G. (2000). Measures of reliability in sports medicine and science. Sports Med..

[62] Hedges L., Olkin I. (1985). Statistical Methods for Meta-Analysis.

[63] Hopkins W.G. (2002). A Scale of Magnitudes for Effect Statistics. http://www.sportsci.org/resource/stats/index.html.

[64] Garhammer J. (1993). A review of power output studies of olympic and powerlifting: Methodology, performance prediction, and evaluation tests. J. Strength Cond. Res..

[65] Enoka R.M. (1979). The pull in olympic weightlifting. Med. Sci. Sports.

[66] Thomas C., Jones P.A., Rothwell J., Chiang C.Y., Comfort P. (2015). An investigation into the relationship between maximum isometric strength and vertical jump performance. J Strength Cond. Res..

[67] James L.P., Beckman E.M., Kelly V.G., Haff G.G. (2016). The neuromuscular qualities of higher and lower-level mixed martial arts competitors. Int. J. Sports Physiol. Perform..

[68] Talpey S.W., Young W.B., Beseler B. (2016). Effect of instructions on selected jump squat variables. J. Strength Cond. Res..

[69] Thomas C., Comfort P., Jones P.A., Dos’Santos T. (2016). A comparison of isometric mid-thigh pull strength, vertical jump, sprint speed, and change of direction speed in academy netball players. Int. J. Sports Physiol. Perform..

