# Supplementary material for: Relationships between Isometric Force-Time Characteristics and Dynamic Performance
Source: Sports (Basel). 2017 Sep 13;5(3):68. doi: 10.3390/sports5030068 (PMC5968956; doi:10.3390/sports5030068)
Supplement: Supplementary file 1 [file sports-05-00068-s001.pdf]

Supplementary Materials: Relationships between Isometric Force-Time Characteristics and Dynamic Performance

Thomas Dos'Santos *, Christopher Thomas, Paul Comfort, John J. McMahon and Paul A. Jones

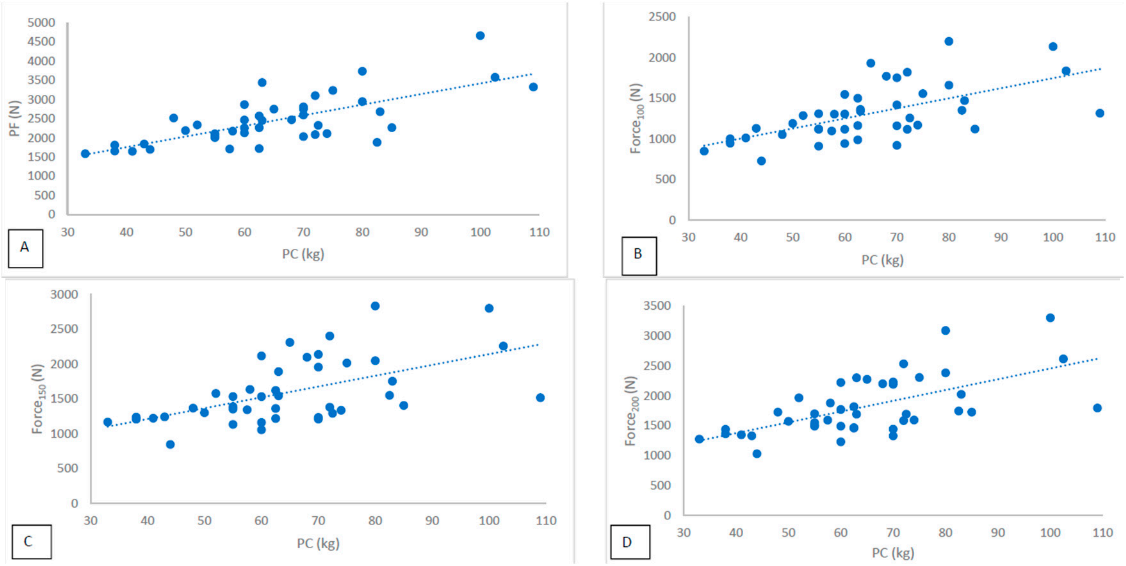

Figure S1. Spearman's correlation between (**A**) PC and PF; (**B**) PC and $Force_{100}$; **(C)** PC and $Force_{150}$; **(D)** PC and $Force_{200}$.

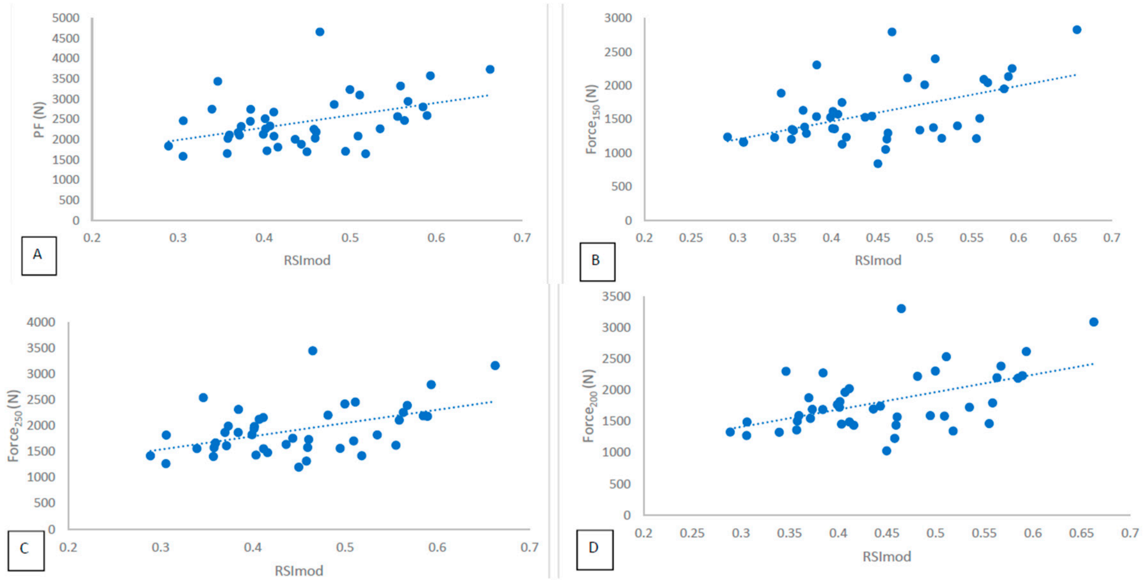

Figure S2. Spearman's correlation between (A) RSImod and PF; (B) RSImod and $Force_{150}$; (C) RSImod and $Force_{250}$; (D) RSImod and $Force_{200}$.

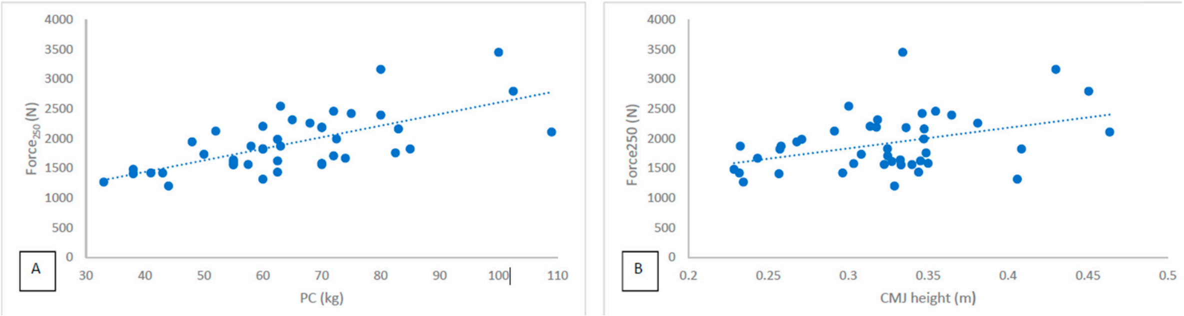

Figure S3. Spearman's correlation between (**A**) PC and $Force_{250}$; (**B**) CMJ height and $Force_{250}$.
